# Integrated Health Record Viewers and Reduction in Duplicate Medical Imaging: Retrospective Observational Analysis

**DOI:** 10.2196/32168

**Published:** 2022-05-20

**Authors:** Yingzhe Yuan, Megan Price, David F Schmidt, Merry Ward, Jonathan Nebeker, Steven Pizer

**Affiliations:** 1 Department of Health Law, Policy & Management, School of Public Health Boston University Boston, MA United States; 2 Partnered Evidence-Based Policy Resource Center Boston Veterans Affairs Healthcare System Boston, MA United States; 3 Veterans Health Administration Office of Health Informatics Department of Veterans Affairs Washington DC, DC United States; 4 School of Medicine Oregon Health and Science University Portland, OR United States; 5 School of Medicine University of Utah Salt Lake City, UT United States

**Keywords:** health informatics, duplicate medical imaging, health record viewer, health care system, health care, health records, electronic health records, health information exchange

## Abstract

**Background:**

Health information exchange and multiplatform health record viewers support more informed medical decisions, improve quality of care, and reduce the risk of adverse outcomes due to fragmentation and discontinuity in care during transition of care. An example of a multiplatform health record viewer is the VA/DoD Joint Longitudinal Viewer (JLV), which supports the Department of Veterans Affairs (VA) and Department of Defense (DoD) health care providers with read-only access to patient medical records integrated from multiple sources. JLV is intended to support more informed medical decisions such as reducing duplicate medical imaging when previous image study results may meet current clinical needs.

**Objective:**

We estimated the impact of provider usage of JLV on duplicate imaging for service members transitioning from the DoD to the VA health care system.

**Methods:**

We conducted a retrospective cross-sectional study in fiscal year 2018 to examine the relationship between providers’ use of JLV and the likelihood of ordering duplicate images. Our sample included recently separated service members who had a VA primary care visit in fiscal year 2018 within 90 days of a DoD imaging study. Patients who received at least one imaging study at VA within 90 days of a DoD imaging study of the same imaging mode and on the same body part are considered to have received potentially duplicate imaging studies. We use a logistic regression model with “JLV provider” (providers with 1 or more JLV audits in the prior 6 months) as the independent variable to estimate the relationship between JLV use and ordering of duplicate images. Control variables included provider image ordering rates in the prior 6 months, provider type, patient demographics (age, race, gender), and clinical characteristics (Elixhauser comorbidity score).

**Results:**

Providers known to utilize JLV in the prior 6 months order fewer duplicate images relative to providers not utilizing JLV for similar visits over time (odds ratio 0.44, 95% CI 0.24-0.78; *P*=.005). This effect is robust across multiple specifications of linear and logistic regression models. The provider’s practice pattern of ordering image studies and the patient’s health status are powerful confounders.

**Conclusions:**

This study provides evidence that adoption of a longitudinal viewer of health records from multiple electronic health record systems is associated with a reduced likelihood of ordering duplicate images. Investments in health information exchange systems may be effective ways to improve the quality of care and reduce adverse outcomes for patients experiencing fragmentation and discontinuity of care.

## Introduction

Health information exchange (HIE) allows health care providers and patients to access and share patient-level electronic health information between different health care settings [1-3]. When health information such as radiology reports, laboratory results, and drug allergy history is shared, HIE helps ensure the safety of patients and improve clinic efficiency [4]. Adoption of HIE has the potential to address the Institute of Medicine’s quality aims [5] and produce substantial financial value [6]. Previous research linked the use of electronic health record viewers or HIE participation to improved health care quality measures such as higher patient satisfaction or lower readmission and duplicate diagnostic imaging study rates. A recent study by Legler et al [7] demonstrated that providers’ early adoption of a longitudinal health record viewer was related to patients more likely reporting that providers were knowledgeable of their medical history. In another recent work, Chen et al [8] found that hospitals’ participation in HIE was associated with a reduction in 30-day readmission rates in Florida. Bailey et al [9] found that use of HIE was associated with a decreased probability of ordering repeated diagnostic imaging in the emergency evaluation of back pain. For patients transitioning between health care systems, fragmentation and discontinuity in care increase the risk of adverse health outcomes [10]. Providers’ access to patient-level medical records from multiple health care settings may support more informed medical decisions, improve quality of care, enhance care coordination, and reduce risks of adverse outcomes due to fragmentation.

Minimizing orders for unnecessary duplicate medical image studies is important for improving health care efficiency, reducing unnecessary time burdens on patients, and attenuating adverse health outcomes caused by excessive medical radiation, such as increased risk of cancer [10-13]. However, there is little evidence regarding the impact of HIE on duplicate imaging. Vest et al [14] found that the use of an HIE system to access previous patient information was associated with a reduction in repeated medical imaging, but the study was limited by its setting in 11 counties in New York and was unable to adjust for potential confounders at the provider level. In this paper, we estimate the impact of provider usage of integrated health record viewers on the ordering of duplicate imaging for patients receiving health care in multiple settings.

An example of integrated health record viewers is the Joint Longitudinal Viewer (JLV), formerly known as the Joint Legacy Viewer (version 2.2). As a web-based graphical user interface, JLV supports the Department of Veterans Affairs (VA) and Department of Defense (DoD) health care providers with an integrated, read-only view of health data from the VA and DoD systems as well as VA community partners [7]. Released on October 1, 2014, JLV has been used by an increasing number of providers to view noncomputable patient-level health information such as vital signs, physician notes, medications, allergy, immunization, and radiology records [15]. The integrated viewer allows providers to access a complete set of the patient’s previous medical images and therefore has the potential to reduce the frequency of duplicate medical image studies.

## Methods

### Study Design

We conducted a retrospective cross-sectional study in fiscal year 2018 to examine the relationship between provider use of JLV and the ordering of potentially duplicate image studies. The analysis compared duplicate imaging ordered by JLV-using and non–JLV-using providers of VA outpatient primary care visits in fiscal year 2018 for recently separated service members. We conducted the study for VA quality improvement and program evaluation purposes, and therefore, the study was exempt from Institutional Review Board review.

### Participants and Setting

Recently separated service members who had at least one VA primary care visit in fiscal year 2018 within 90 days of an imaging study conducted at DoD were eligible to be included in the sample. We excluded VA primary care visits that were compensation and pension exams or not provided by physicians, physician assistants, or nurse practitioners. We also excluded DoD imaging studies if the primary diagnosis was cancer because duplicate diagnostic images were likely to be clinically appropriate and recommended by providers for patients with cancer. Patients who received at least one imaging study at VA within 90 days of a DoD imaging study using the same imaging mode and on the same body part were considered to have received potentially duplicate imaging studies [11].

### Measures

VA clinic stop codes (322, 323, and 350) were used to identify outpatient primary care visits. Compensation and Pension exams were identified using the secondary stop code (450) and the appointment type (Compensation and Pension) and were excluded from the VA primary care visits. Audit logs acquired from the JLV system were assessed to determine a provider’s JLV utilization during a specific VA primary care visit and the provider's JLV utilization history over the 6 months prior to the visit.

The independent variable “JLV encounter” indicated whether a JLV audit was linked to the patient on the primary care visit date. The independent variable “JLV provider” indicated whether the provider had 1 or more JLV audits in the 6 months prior to the visit date. Endogeneity is likely to be a problem in the estimation of the association between “JLV encounter” and duplicate imaging because unobserved confounders such as patient complexity are related to both JLV use and ordering duplicate image studies during the primary care visit. We estimated the direct (proxy) relationship between “JLV provider” and duplicate imaging to deal with the potential endogeneity problem. We also used a 2-stage statistical model by using JLV providers as an instrumental variable to estimate the causal relationship between JLV encounter and duplicate imaging. The categorization of JLV providers was based on the provider’s prior interactions with other patients and indicated the provider’s propensity to view health records through JLV. Thus, this variable was independent of the observed and unobserved characteristics of the patient under study. This is especially true in the VA setting where patients are arbitrarily assigned to primary care providers. Current Procedural Terminology codes indicating imaging procedures were categorized by mode and body part to compare VA and DoD imaging records and identify potential duplicate images. Following Vest et al [14], the dependent variable was coded as “duplicate image” if an imaging study ordered during the VA primary care visit was of the same mode and the same body part as a DoD imaging study for the patient within 90 days prior to the VA visit date. Covariates included the provider’s rate of ordering images during previous primary care visits with other patients over the 6 months prior to the VA visit date, provider type (physicians and physician assistants/nurse practitioners), patient demographics (age, gender, and race), clinical characteristics (Elixhauser comorbidity score), and fiscal month (October 2017 to September 2018).

### Statistical Analyses

Descriptive statistics were calculated to explore the distributions of duplicate image ordering, the provider’s rate of ordering images in the prior 6 months, the patient’s Elixhauser comorbidity score, and other covariates. In our primary statistical model, we used a logistic regression to estimate the relationship between the provider’s JLV use in the prior 6 months (yes/no) and duplicate imaging (yes/no). In an alternative specification, we used instrumental variables to focus on JLV use in the actual primary care visit. To deal with the potential endogeneity problem that unobserved patient characteristics might confound that relationship, we used a 2-stage residual inclusion (2SRI) logistic regression model to estimate the relationship between the provider’s JLV use during the primary care visit (yes/no) and the ordering of duplicate imaging studies (yes/no) with JLV provider as the instrumental variable.

More formally, we wished to estimate the relationship between duplicate imaging and use of JLV during the primary care visit, controlling for potential confounders, including provider imaging rate in the prior 6 months, provider type (physician or physician assistant/nurse practitioner), patient age, gender, Elixhauser comorbidity risk score, time (month), and facility (VA Medical Center) (equation 1). This model could produce biased estimates because the decision to use JLV during the visit could be simultaneously determined with the decision to order a duplicate imaging study due to unobserved confounding factors such as patient complexity. To address this problem, we estimated the first stage model, which related JLV use during the visit to the provider’s JLV use history (the JLV provider variable). Then, we estimated the second stage model, which related duplicate imaging to JLV use during the visit. Anscombe residuals (X_µe_) calculated from the first stage were included in the second stage model because 2SRI models with Anscombe residuals generate less biased estimates for rare outcomes compared to 2SRI models with other forms of residuals [16]. Bootstrapping was used to improve the estimation of standard errors.

2SRI logistic regression model: Y = f(X_e_β_e_ + X_o_β_o_) + X_µ_β_µ_ + ε **(1)**

Y: Provider ordering duplicate imaging

X_e_: JLV encounter, endogenous

X_o_: Provider imaging rate in prior 6 months, provider type (physician or physician assistant/nurse practitioner), patient age, gender, Elixhauser comorbidity risk score, time (month), and facility (VA Medical Center)

X_µ_: Unobserved confounding factor such as patient complexity

*ε*: Residual

First stage: X_e_ = Wα + X_µ_β_µ_
**(2)**

W: The instrument of JLV provider and observed exogenous variables

Second stage: Y = f(X_e_β_e_ + X_o_β_o_ + X_µe_β_µ_) + ε **(3)**

X_µe_: Anscombe residual calculated from the first stage model estimates

We tested the robustness of the result by using different model specifications, including ordinary least squares models on the relationship between JLV provider and duplicate imaging and linear 2-stage least squares models on the relationship between JLV encounter and duplicate imaging using JLV provider as an instrumental variable. We used Stata 15.1 (StataCorp LLC) to conduct the statistical analysis.

## Results

**Figure 1:**
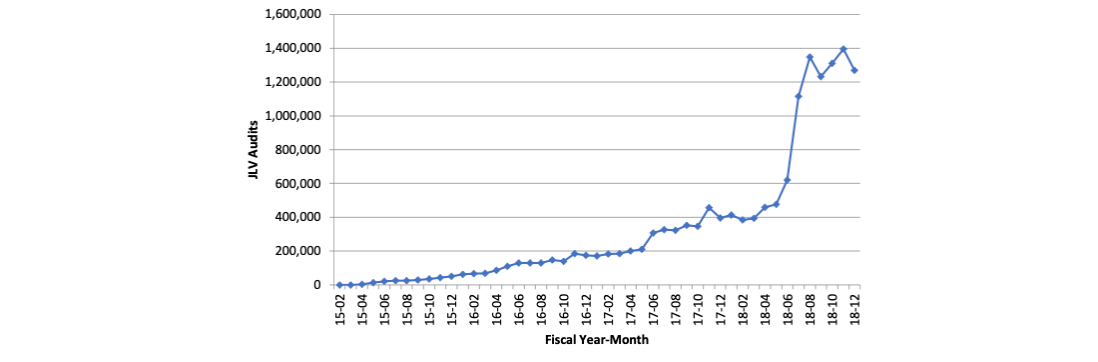
Joint Longitudinal Viewer use growth. JLV: Joint Longitudinal Viewer.

Overall, JLV use has increased since fiscal year 2015. Rapid growth of monthly JLV audits was observed in fiscal year 2018 (Figure 1). Table 1 shows that the duplicate imaging rate among non–JLV encounters was 7.8% (34/435) and the duplicate imaging rate among JLV encounters was 7.9% (36/457). However, a direct comparison of these rates may be a biased estimate of the effect of JLV use owing to the endogeneity problem discussed above. The duplicate imaging rates were 11.2% (34/305) and 6.1% (36/587) among the non–JLV provider and JLV provider groups, respectively. Unlike the first comparison, this one should not be biased by uncontrolled differences in patient characteristics. Of the 892 unique patient-provider encounters in our analytic sample, 588 (65.9%) were males and the average age was 34.3 years, 512 (57.4%) patients were White, 228 (25.6%) were Black, and 152 (17%) were of other races. On average, patients had an Elixhauser comorbidity score of 1.2. Among the providers of these encounters, 336 (37.7%) were physicians and 556 (62.3%) were physician assistants/nurse practitioners. Providers had an average image ordering rate of 17.5% in the prior 6 months. Average patient and provider characteristics were not significantly different between the JLV encounter and non–JLV encounter groups or between the JLV provider and non–JLV provider groups (Table 1).

**Table 1:** Characteristics of the recently separated service members receiving Veterans Affairs primary care in fiscal year 2018 and characteristics of the related primary care providers (by Joint Longitudinal Viewer Encounter and Joint Longitudinal Viewer Provider).

Characteristics				Non–JLV^a^ encounter (n=435)		JLV encounter (n=457)		Non–JLV provider (n=305)		JLV provider (n=587)		Overall (N=892)
**Patient characteristics**												
	Gender (male), n (%)		298 (68.5)		290 (63.5)		208 (68.2)		380 (64.7)		588 (65.9)	
	Age (years), mean		34.2		34.3		34.5		34.1		34.3	
	**Race, n (%)**											
		White	243 (55.9)		269 (58.9)		164 (53.8)		348 (59.3)		512 (57.4)	
		Black or African American	114 (26.2)		114 (24.9)		85 (27.9)		143 (24.4)		228 (25.6)	
		Other	78 (17.9)		74 (16.2)		56 (18.4)		96 (16.4)		152 (17)	
	Elixhauser comorbidity score (mean)		1.16		1.28		1.21		1.23		1.22	
**Provider characteristics**												
	Provider history rate of ordering imaging studies (mean)		17.8		17.2		18		17.3		17.5	
	**Provider type, n (%)**											
		Physician	169 (38.9)		167 (36.5)		138 (45.3)		198 (33.7)		336 (37.7)	
		Physician assistant/Nurse practitioner	266 (61.1)		290 (63.5)		167 (54.8)		389 (66.3)		556 (62.3)	

^a^JLV: Joint Longitudinal Viewer.

In our primary analysis with provider history of JLV utilization as the independent variable, after controlling for patient and provider characteristics, provider JLV use was significantly associated with a reduced likelihood (odds ratio [OR] 0.44, 95% CI 0.24-0.78; *P*=.005; average incremental effect=–0.05) of ordering duplicate image studies. Provider history of ordering images and patient Elixhauser comorbidity scores were strong confounders of the relationship between JLV use and duplicate imaging. Providers with high rates of ordering images in the prior 6 months were more likely to order duplicate images (OR 4.15, 95% CI 1.86-9.25; *P*=.001). Patient Elixhauser comorbidity scores of 3 or more were significantly associated with a reduced likelihood of receiving duplicate imaging services (OR 0.15, 95% CI 0.04-0.52; *P*=.003) (Table 2). In a logistic regression model, the average incremental effect is a nonlinear function of the coefficients and values of other explanatory variables [17]. Although the average incremental effect is easier to interpret than the OR, the statistical significance of the average incremental effect does not necessarily correspond to the significance of the coefficient or OR. As a result, the significance of the average incremental effect is not reported above.

In the 2SRI analysis, the results of our first stage model (Table 3) indicated that past use of JLV was strongly predictive of use of JLV by the provider during the primary care encounter (OR 1.43, 95% CI 1.05-1.81; *P*<.001). In a test of the coefficient on the instrument, a Cragg-Donald Wald F statistic greater than 10 indicates that the instrument is strong enough [17]. In a linear version of the first stage model, the strength of the instrument was tested (Cragg-Donald Wald F=43.68; *P*<.001). The Cragg-Donald Wald F statistic of 43.68 is greater than 10 and suggests that provider past use of JLV was a strong instrument. This is a necessary condition for JLV provider to serve as an instrumental variable for JLV encounter in our 2-stage specifications.

In the analysis assessing the relationship between JLV encounter and duplicate imaging with JLV provider as an instrumental variable, provider use of JLV was significantly associated with a reduction (OR 0.08, 95% CI 0.01-0.81; *P*=.03; average incremental effect=–0.16) in the likelihood of ordering duplicate images, controlling for patient and provider characteristics, time effects, and facility random effects (Table 4). Provider history of ordering images and patient Elixhauser comorbidity scores were strong confounders of the relationship between JLV use and duplicate imaging. Providers with high rates of ordering image studies in the prior 6 months were more likely to order duplicate images (OR 3.93, 95% CI 1.42-10.94; *P*=.009) compared to providers with low rates of ordering medical image studies. Patient Elixhauser comorbidity scores of 3 or more were significantly associated with a reduced likelihood of receiving duplicate imaging procedures (OR 0.16, 95% CI 0.04-0.57; *P*=.005) (Table 4).

Our main finding that JLV use had a significant effect on reducing duplicate imaging was robust using different model specifications, including 2-stage least squares models estimating the association between JLV encounter and duplicate imaging (see Table S2 in Multimedia Appendix 1).

**Table 2:** The impact of provider use of Joint Longitudinal Viewer in the prior 6 months of outpatient primary care visits on provider ordering of duplicate images.

Characteristics					Odds ratio (95% CI)^a^			*P* value
**Provider characteristics**								
	Joint Longitudinal Viewer provider			0.44 (0.24-0.78)			.005	
	**Provider history of ordering imaging studies (quartiles)**							
		1	Ref^b^			Ref		
		2	0.87 (0.33-2.32)			.78		
		3	2.73 (1.23-6.06)			.01		
		4	4.15 (1.86-9.25)			.001		
	**Provider type**							
		Physician	Ref			Ref		
		Physician assistant/Nurse practitioner	1.32 (0.75-2.32)			.34		
**Patient characteristics**								
	**Gender**							
		Female	Ref			Ref		
		Male	1.68 (0.89-3.17)			.11		
	**Age (years)**							
		<30	Ref			Ref		
		30-39	2.19 (1.17-4.11)			.01		
		40-49	0.78 (0.35-1.73)			.54		
		≥50	1.27 (0.46-3.56)			.65		
	**Race**							
		White	Ref			Ref		
		Black or African American	1.09 (0.56-2.13)			.80		
		Other	1.63 (0.82-3.21)			.16		
	**Elixhauser comorbidity score**							
		0	Ref			Ref		
		1	0.44 (0.23-0.83)			.01		
		2	0.40 (0.18-0.90)			.03		
		3 and above	0.15 (0.04-0.52)			.003		

^a^The odds ratio and 95% CIs are estimated from the logistic regression model controlling for all variables shown in the table as well as facility (random effects) and fiscal month.

^b^Ref indicates baseline in the analysis.

**Table 3:** The impact of provider use of Joint Longitudinal Viewer during outpatient primary care visits on provider ordering of duplicate images (stage 1 full output).^a^

Joint Longitudinal Viewer encounter (first stage)				Odds ratio (95% CI)		*P* value
Joint Longitudinal Viewer provider				1.43 (1.05 to 1.81)		<.001
**Provider characteristics**						
	**Provider history of ordering imaging studies (quartiles)**					
		1	Ref^b^		Ref	
		2	–0.18 (–0.63 to 0.28)		.45	
		3	0.30 (–0.17 to 0.78)		.21	
		4	–0.19 (–0.67 to 0.30)		.46	
	**Provider type**					
		Physician	Ref		Ref	
		Physician assistant/Nurse practitioner	–0.06 (–0.42 to 0.30)		.75	
**Patient characteristics**						
	**Gender**					
		Female	Ref		Ref	
		Male	–0.20 (–0.56 to 0.16)		.28	
	**Age (years)**					
		<30	Ref		Ref	
		30-39	0.03 (–0.38 to 0.43)		.90	
		40-49	0.10 (–0.34 to 0.55)		.64	
		≥50	0.37 (–0.30 to 1.03)		.28	
	**Race**					
		White	Ref		Ref	
		Black or African American	–0.30 (–0.70 to 0.11)		.15	
		Other	–0.29 (–0.75 to 0.18)		.22	
	**Elixhauser comorbidity score**					
		0	Ref		Ref	
		1	–0.01 (–0.40 to 0.39)		.98	
		2	0.29 (–0.19 to 0.77)		.23	
		3 and above	0.13 (–0.37 to 0.64)		.61	
	**Fiscal month**					
		1	Ref		Ref	
		2	0.15 (–0.59 to 0.90)		.69	
		3	–0.47 (–1.26 to 0.31)		.24	
		4	–0.63 (–1.42 to 0.17)		.12	
		5	–0.14 (–0.97 to 0.68)		.73	
		6	–0.49 (–1.27 to 0.29)		.22	
		7	0.62 (–0.15 to 1.39)		.12	
		8	0.61 (–0.10 to 1.33)		.09	
		9	0.65 (–0.07 to 1.37)		.08	
		10	0.59 (–0.16 to 1.35)		.12	
		11	0.68 (–0.10 to 1.46)		.09	
		12	0.77 (–0.09 to 1.63)		.08	
Cons^c^				–1.11 (–1.88 to –0.33)		.005

^a^Average incremental effects are estimated from the 2-stage residual inclusion logistic regression controlling for all variables shown in the table.

^b^Ref indicates baseline in the analysis.

^c^Cons: Constant term in the regression.

**Table 4:** The impact of provider use of Joint Longitudinal Viewer during outpatient primary care visits on provider ordering of duplicate images (Stage 2 full output).^a^

Duplicate imaging (second stage)				Odds ratio (95% CI)		*P* value
Joint Longitudinal Viewer encounter				0.08 (0.01-0.81)		.03
Anscombe residual				3.16 (1.29-7.79)		.01
**Provider characteristics**						
	**Provider history of ordering imaging studies (quartiles)**					
		1	Ref^b^		Ref	
		2	0.83 (0.23-3.07)		.78	
		3	3.11 (1.18-8.22)		.02	
		4	3.93 (1.42-10.94)		.009	
	**Provider type**					
		Physician	Ref		Ref	
		Physician assistant/Nurse practitioner	1.24 (0.61-2.51)		.56	
**Patient characteristics**						
	**Gender**					
		Female	Ref		Ref	
		Male	1.49 (0.72-3.08)		.28	
	**Age (years)**					
		<30	Ref		Ref	
		30-39	2.28 (0.98-5.34)		.06	
		40-49	0.87 (0.32-2.42)		.79	
		≥50	1.50 (0.38-5.93)		.57	
	**Race**					
		White	Ref		Ref	
		Black or African American	1.03 (0.48-2.18)		.95	
		Other	1.57 (0.65-3.80)		.32	
	**Elixhauser comorbidity score**					
		0	Ref		Ref	
		1	0.43 (0.20-0.91)		.03	
		2	0.43 (0.15-1.21)		.11	
		3 and above	0.16 (0.04-0.57)		.005	
	**Fiscal month**					
		1	Ref		Ref	
		2	1.41 (0.00-602.30)		.91	
		3	1.31 (0.00-542.59)		.93	
		4	1.43 (0.00-639.45)		.91	
		5	0.75 (0.00-290.28)		.93	
		6	1.10 (0.00-523.39)		.98	
		7	3.15 (0.01-1539.80)		.72	
		8	1.77 (0.01-819.76)		.86	
		9	4.11 (0.01-1699.58)		.65	
		10	4.41 (0.01-2011.70)		.64	
		11	1.96 (0.01-762.17)		.83	
		12	5.02 (0.01-2177.41)		.60	
Cons^c^				0.05 (0.00-22.75)		.34

^a^Average incremental effects are estimated from the 2-stage residual inclusion logistic regression controlling for all variables shown in the table.

^b^Ref indicates baseline in the analysis.

^c^Cons: Constant term in the regression.

## Discussion

This study using national data from VA and DoD found that providers who viewed integrated patient health records from multiple settings were less likely to order potentially duplicate imaging studies for patients who had prior imaging studies conducted within 90 days. Based on results from our primary analysis, providers with a history of using JLV were 5 percentage points less likely to order duplicate images during a VA primary care visit for recently separated service members compared to providers who did not have a history of using JLV. Using the JLV provider as an independent variable, we were able to reduce potential endogeneity due to unobserved confounders that were associated with both JLV use during the primary care visit and ordering of duplicate images.

Our results were consistent with previous findings that use of HIE systems was associated with a reduction in repeat imaging studies [14] and that a longitudinal viewer of patient records from multiple sources was related to more positive patient experiences of care [7]. Our analysis had the added advantage of including provider-level variables, primarily a provider history of ordering images, which appeared to be a strong confounder. Access to national-level VA and DoD data also enabled the study to focus on images ordered for recently separated service members, who were transitioning between health care delivery systems and may be particularly likely to benefit from investments in integrated health information viewers or HIE systems.

Our study has several limitations. First, we focused on VA primary care visits and images within the 90-day follow-up period of a DoD image and therefore were unable to capture duplicate images in other settings such as community-based clinics. Further research could examine duplicate image studies ordered during different types of outpatient and inpatient encounters to improve the generalizability of the results. Second, limited by the administrative data source, we could not determine whether the identified duplicate imaging procedures were unnecessary. In some cases, providers may need to examine repeat image studies for serial changes in disease status or order follow-up imaging studies based on recommendations in the patient’s previous imaging reports. Thus, some of the duplicate image studies we identified might have been clinically appropriate. We mitigated this limitation by excluding patients with cancer diagnoses and ensuring the consistency of the definition of duplicate imaging studies among the providers who used and did not use JLV. Third, restricted by data access, we could not adjust for HIE through another widely used health information viewer, VistaWeb, which was recently decommissioned. We tried to overcome this limitation by focusing on VA primary care visits in fiscal year 2018—the year when we observed rapid growth in JLV utilization after the VA’s transition from VistaWeb to JLV. Fourth, our result was not robust when we changed the definition of JLV provider to providers with 10 or more JLV audits in the 6 months prior to the VA primary care visit, suggesting the heterogeneity of JLV benefits by frequency of use.

Organizational fragmentation and discontinuity of care have been linked to increased costs and adverse outcomes in VA and other health care settings [6]. Our findings suggest that the use of a longitudinal viewer of health records from multiple electronic health record sources has the potential to alleviate patient time burden, reduce adverse health effects of radiation, and decrease costs resulting from unnecessary duplicate imaging procedures. Health systems outside the VA could also consider investments in health information viewers or HIE technology to reduce the deleterious effects of fragmentation.

In conclusion, this study provides evidence that adoption of a longitudinal viewer of health records from multiple electronic health record systems is associated with a reduced likelihood of ordering duplicate image studies. In future studies, the association between health information viewers and other types of duplicate medical tests and care coordination metrics such as follow-up of suspicious lung nodules could be investigated to more fully illustrate the impact of HIE on quality and efficiency of care.

## Appendix

Multimedia Appendix 1 Full definitions of covariates and results from sensitivity analyses.

## Abbreviations

2SRI: 2-stage residual inclusion
DoD: Department of Defense
HIE: health information exchange
JLV: Joint Longitudinal Viewer
OR: odds ratio
VA: Veterans Affairs
