# Supplementary material for: Integrated Health Record Viewers and Reduction in Duplicate Medical Imaging: Retrospective Observational Analysis
Source: JMIR Med Inform. 2022 May 20;10(5):e32168. doi: 10.2196/32168 (PMC9166659; doi:10.2196/32168)
Supplement: Multimedia Appendix 1 [file medinform_v10i5e32168_app1.docx]

**Multimedia Appendix 1. Full definitions of covariates and results from sensitivity analyses.**

*Joint Longitudinal Viewer (JLV) Encounter*

JLV encounter is a binary variable indicating whether a JLV audit was linked to the patient on the VA primary care visit date.

*JLV provider*

JLV provider is a binary variable indicating whether the provider had 1 or more JLV audits in the six months prior to the Veterans Health Affairs (VHA) primary care visit date. For patients having multiple records with different providers on a primary care visit day, the provider who had the most JLV audits (excluding JLV audits >=2,500) in the prior six months was identified.

*Duplicate Imaging*

The main outcome duplicate imaging is defined as whether an imaging study ordered during the VHA outpatient primary care visit was of the same mode and the same body part as a DoD imaging study for the patient within 90 days prior to the VHA visit date. The imaging modes include computerized tomography (CT) scan, X-ray, magnetic resonance imaging/magnetic resonance angiography (MRI/MRA), Ultrasound and Other; the body parts include abdomen, breast, cardiac, chest, gastrointestinal, head neck, lower extremity, urology, pelvis hip, pregnant uterus, renal, spine, upper extremity, vascular, other, and shoulder.

*Covariates*

We identified a series of patient and provider characteristic covariates to compare the JLV and non-JLV groups. The full definitions of the covariates are listed in Table S1.

| **Table S1. Full Definitions of Covariates** | |
| --- | --- |
| **Variable** | **Definition** |
| Gender | Gender (male; female) |
| Age | Age at VHA primary care outpatient visit date |
| Race | White, Black or African American |
| Elixhauser Comorbidity Score | The number of Elixhauser comorbidities calculated with ICD-10 codes from any outpatient visit or inpatient stay at DoD during the 12 months prior to VHA primary care outpatient visit |
| Provider history of ordering imaging studies | Provider's rate of ordering images during previous primary care visits with other patients over the six months prior to the VHA visit date |
| Provider type | Physicians; Physician assistants/Nurse practitioners(PA/NPs) |

Sensitivity tests

Sensitivity tests included simple ordinary least squares (OLS) and logistic regression models using “JLV encounter” as the independent variable, with or without time and facility effects; 2-stage least squares (2SLS) models with or without time and facility effects; 2-stage residual inclusion (2SRI) models with or without time and facility effects; linear and logistic models with or without time and facility effects using “JLV provider” as the independent variable, and a 2SRI logistic model using a different definition of JLV provider as the instrumental variable. The main coefficients for JLV are summarized and listed in Table S2.

In the OLS and logistic regression models on the relationship between JLV encounter and duplicate imaging, provider use of JLV during the primary care visit was not significantly associated with an increased likelihood of duplicate imaging. The estimation was biased due to the endogeneity problem that some unobserved patient characteristics were related to both providers’ use of JLV during the encounter and the likelihood of ordering a duplicate image. Our main analysis on the relationship between JLV provider and duplicate imaging and the sensitivity analyses with JLV provider as an instrumental variable dealt with the endogeneity problem and generated negative and statistically significant results.

| **Table S2. Summary of JLV effects in OLS and Logistic Regression Models** | | | | | |
| --- | --- | --- | --- | --- | --- |
| *OLS Models on JLV use and Number of duplicate imaging procedures* | | | | | |
| **Model** | **Independent Variable** | **Specification** | **# of obs** | **Coefficient (95% CI)** | ***P* value** |
| 1 | JLV encounter | Simple OLS | 890 | 0.08 (–0.04 to 0.19) | .18 |
| 2 | JLV encounter | OLS with time effects | 890 | 0.07 (–0.05 to 0.19) | .24 |
| 3 | JLV encounter | OLS with time and station fixed effects | 890 | 0.16 (0.02 to 0.3) | .02 |
| 4 | JLV encounter | OLS with IV (2SLS) | 890 | -0.48 (–0.81to 0.15) | .004 |
| 5 | JLV encounter | OLS with IV and time effects | 890 | -0.73 (–1.16 to 0.3) | .001 |
| 6 | JLV encounter | OLS with IV and time and station fixed effects | 890 | -0.84 (–1.52 to 0.15) | .02 |
| 7 | JLV provider | OLS | 890 | -0.18 (–0.3 to 0.06) | .003 |
| 8 | JLV provider | OLS with time effects | 890 | -0.24 (–0.37 to 0.11) | <.001 |
| 9 | JLV provider | OLS with time effects, and station fixed effects | 890 | -0.21 (–0.36 to 0.06) | .007 |
| *Logistic Regression Models on JLV use and ordering of duplicate images* | | | | | |
| **Model** | **Independent Variable** | **Specification** | **# of obs** | **Odds Ratio** | **p-value** |
| 10 | JLV encounter | Simple Logit | 892 | 1.13 (0.68 to 1.89) | .63 |
| 11 | JLV encounter | Logit with time effects | 892 | 1.04 (0.6 to 1.8) | .88 |
| 12 | JLV encounter | Logit with time and station random effects | 892 | 1.09 (0.61 to 1.93) | .77 |
| 13 | JLV encounter | Logit model with IV (2SRI) | 892 | 0.22 (0.02 to 1.97) | .18 |
| 14 | JLV encounter | Logit model with IV (2SRI) and time effects | 892 | 0.2 (0.01 to 2.93) | .24 |
| 15 | JLV encounter | Logit model with IV (2SRI), time and station mixed effects | 892 | 0.08 (0.01 to 0.81) | .03 |
| 16 | JLV provider | Logit model | 892 | 0.55 (0.33 to 0.93) | .03 |
| 17 | JLV provider | Logit model with time effects | 892 | 0.44 (0.24 to 0.78) | .005 |
| 18 | JLV provider | Logit model with time effects and station random effects | 892 | 0.44 (0.24 to 0.78) | .005 |
| 19 | JLV encounter | Logit model with IV (2SRI), time and station mixed effects | 892 | 0.22 (0.02 to 2.27) | .21 |
|  | | | | | |

In the sensitivity analyses using different cut points to define “JLV Provider”, the association between JLV use and a reduced likelihood of ordering duplicate images was not significant if the cut point was greater than 2 audits (see Table S3). This is due to residual provider treatment pattern that confounds the association between JLV use and ordering duplicate images.

| **Table S3. First- and Second- stage Output from 2SRI Logistic Regression Models with Different Definitions of JLV Provider** | | | | | | | | |
| --- | --- | --- | --- | --- | --- | --- | --- | --- |
|  | **First stage** | | | | **Second stage** | | | |
| **Cut point (Audits)** | **Coef.** | **Std. Err.** | **z** | ***P* value** | **Observed Odds Ratio** | **Bootstrap Std. Err.** | **z** | ***P* value** |
| 0 | 1.43 | 0.19 | 7.43 | <.001 | 0.08 | 0.09 | –2.14 | .03 |
| 1 | 1.43 | 0.19 | 7.43 | <.001 | 0.08 | 0.09 | –2.14 | .03 |
| 2 | 1.43 | 0.19 | 7.54 | <.001 | 0.08 | 0.10 | –2.12 | .03 |
| 3 | 1.42 | 0.19 | 7.63 | <.001 | 0.14 | 0.16 | –1.67 | .09 |
| 4 | 1.45 | 0.19 | 7.79 | <.001 | 0.12 | 0.13 | –1.87 | .06 |
| 5 | 1.46 | 0.18 | 7.91 | <.001 | 0.15 | 0.18 | –1.62 | .11 |
| 6 | 1.40 | 0.18 | 7.66 | <.001 | 0.16 | 0.19 | –1.55 | .12 |
| 7 | 1.40 | 0.18 | 7.73 | <.001 | 0.19 | 0.23 | –1.37 | .17 |
| 10 | 1.35 | 0.18 | 7.59 | <.001 | 0.22 | 0.26 | –1.27 | .21 |
| 20 | 1.42 | 0.18 | 8.05 | <.001 | 0.29 | 0.38 | –0.94 | .35 |
| 30 | 1.44 | 0.18 | 8.01 | <.001 | 0.54 | 0.73 | –0.46 | .65 |
| 40 | 1.49 | 0.18 | 8.16 | <.001 | 0.40 | 0.53 | –0.70 | .49 |
